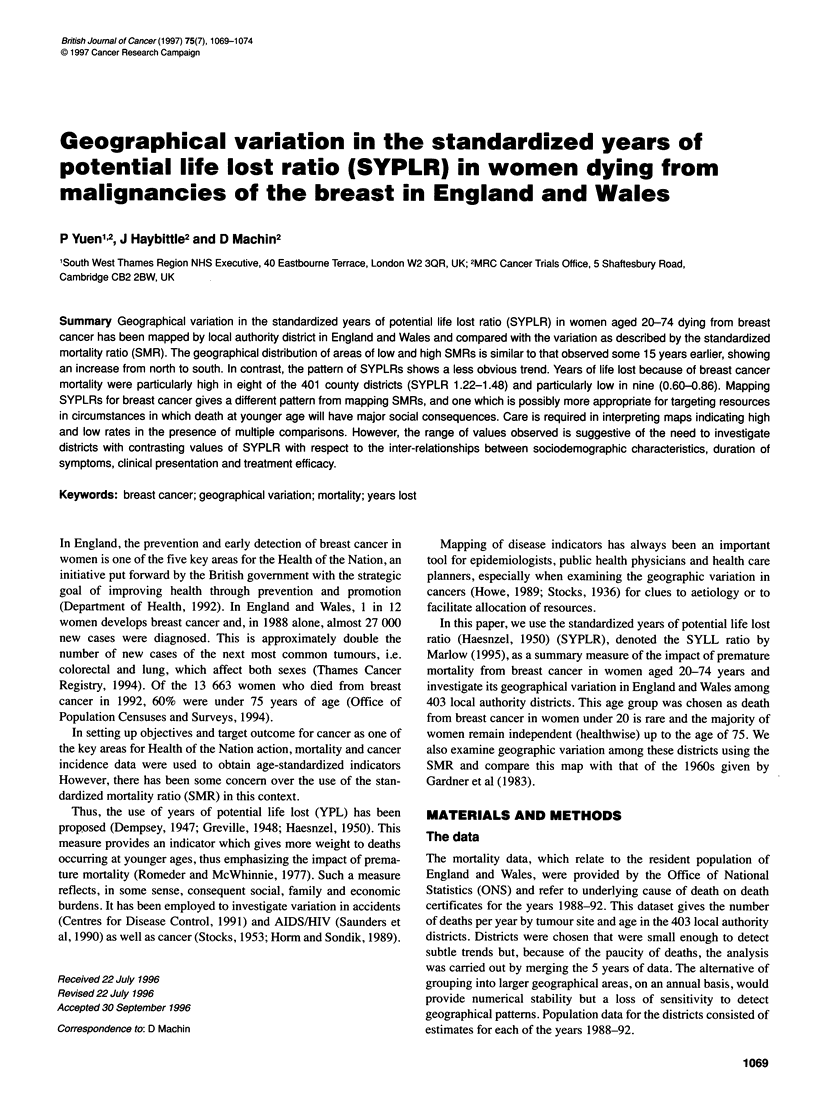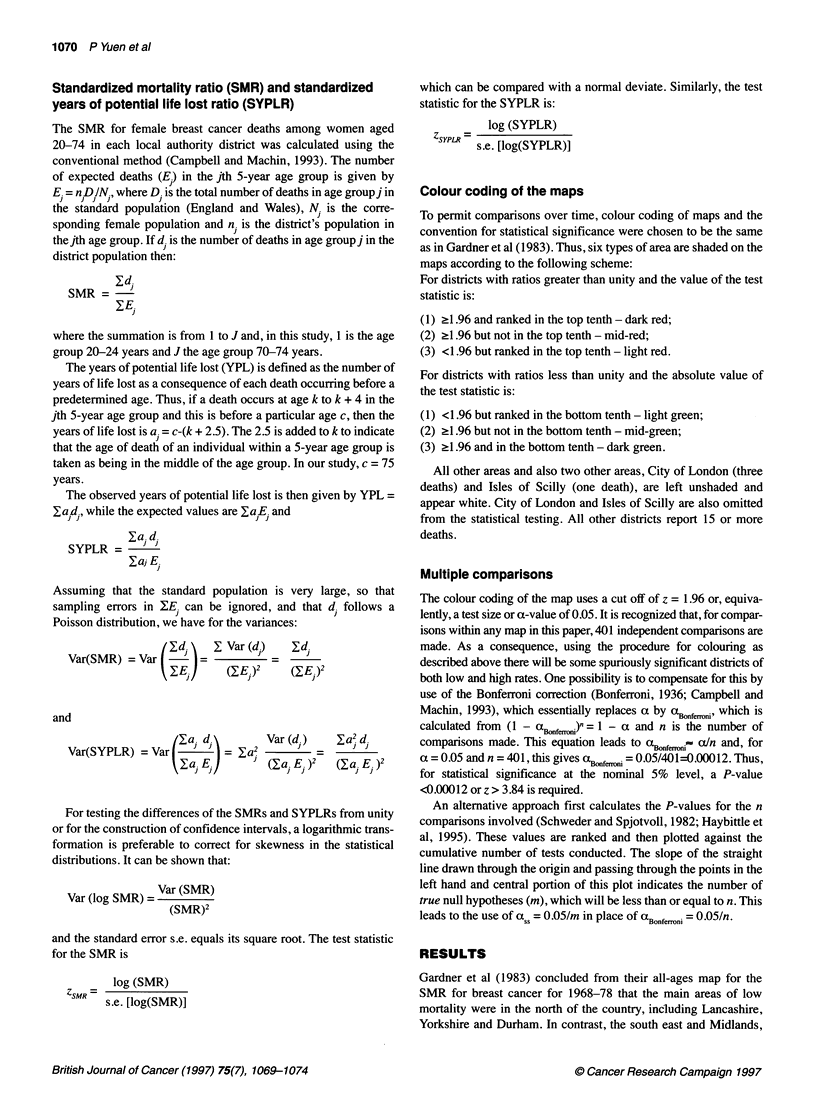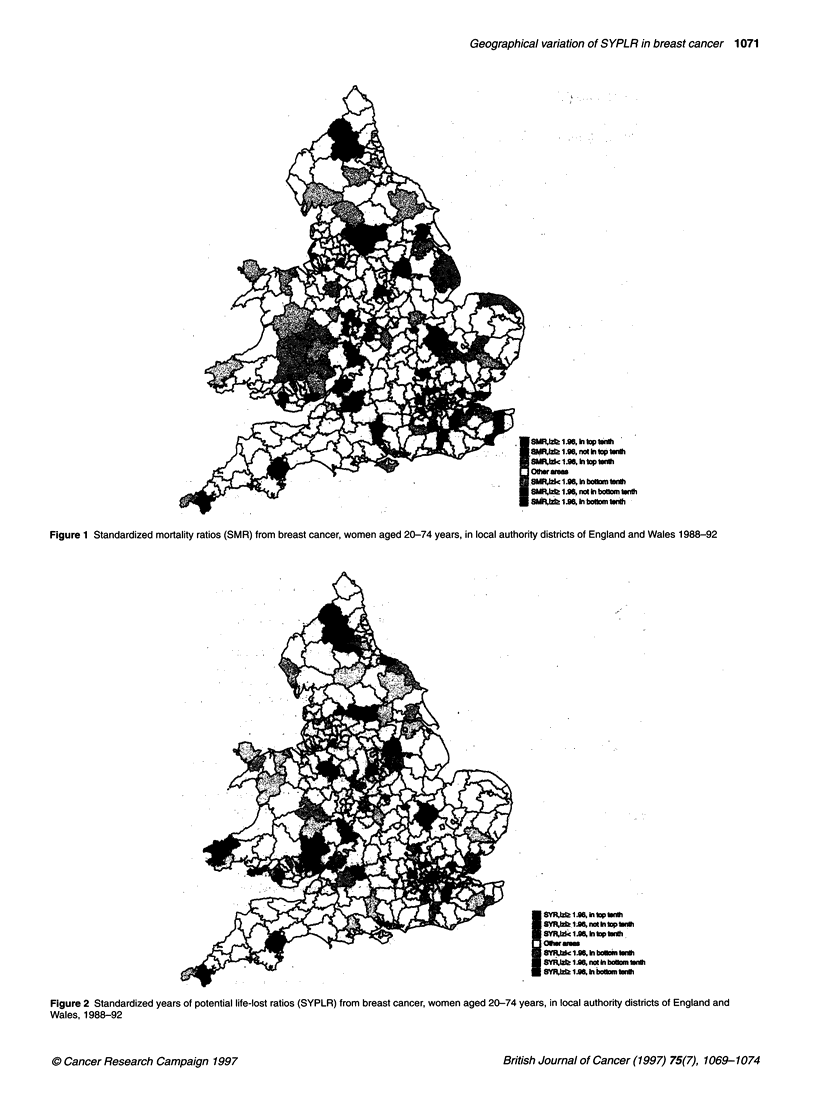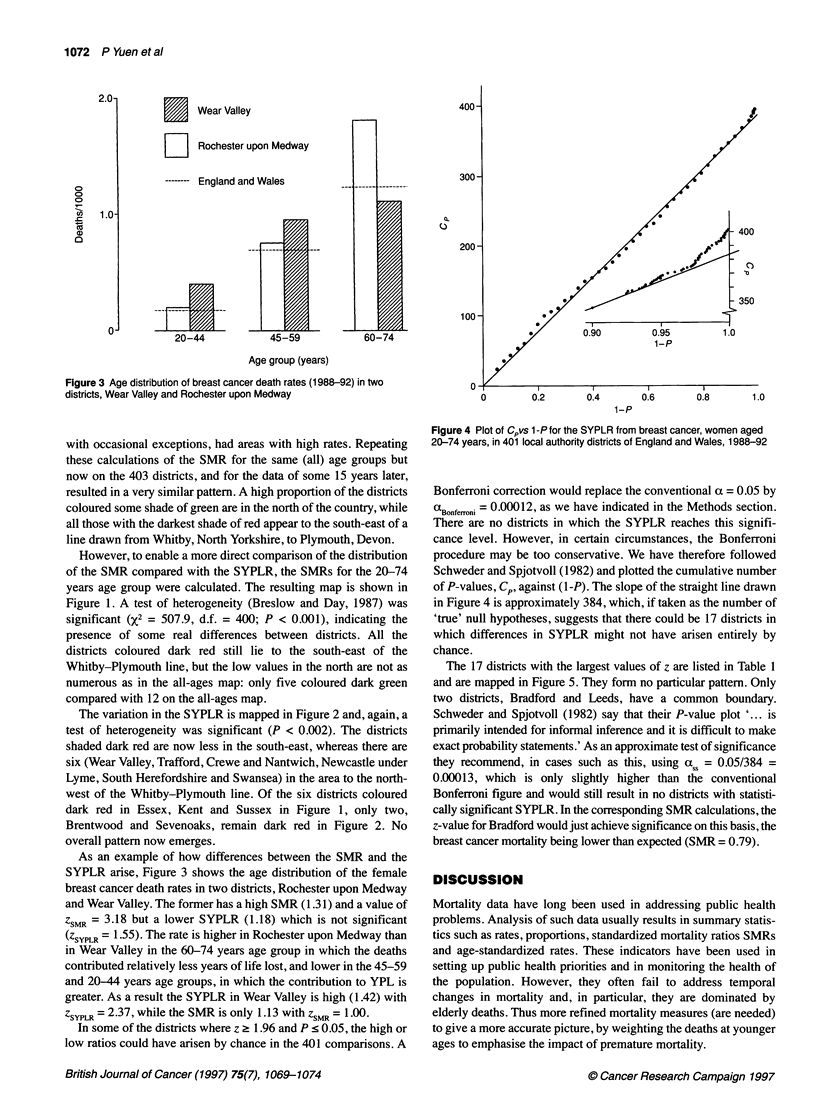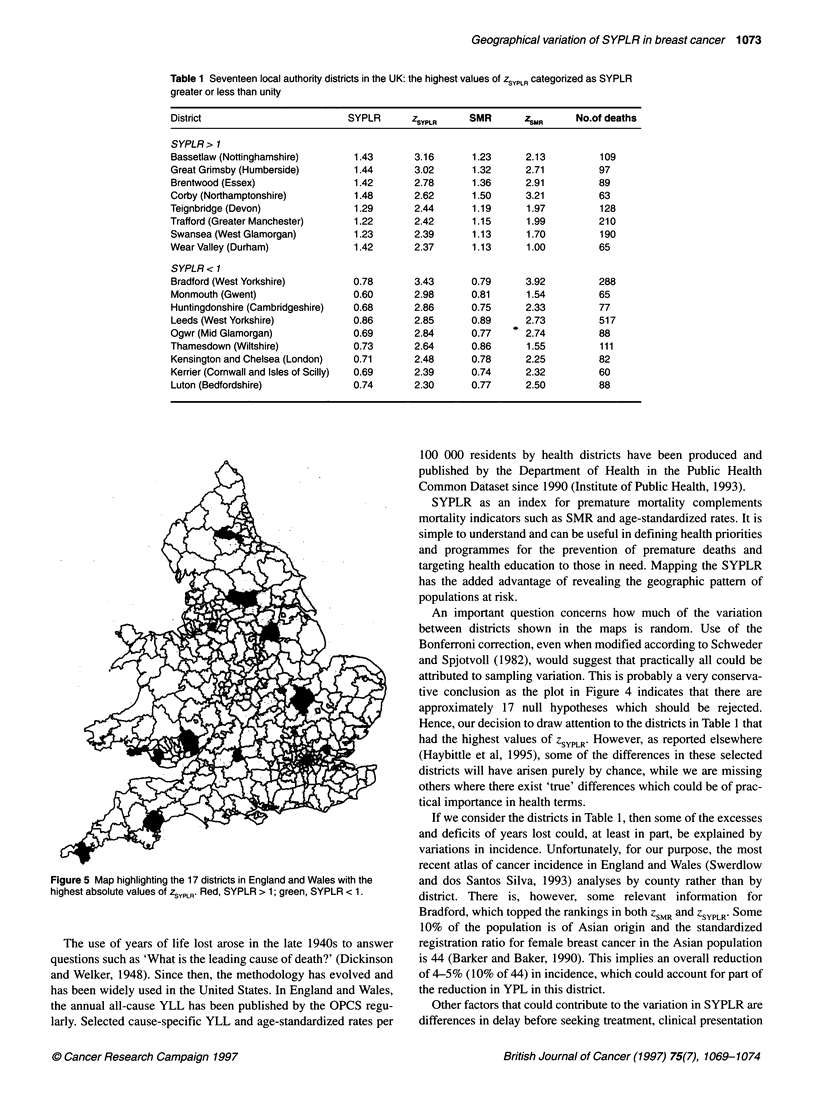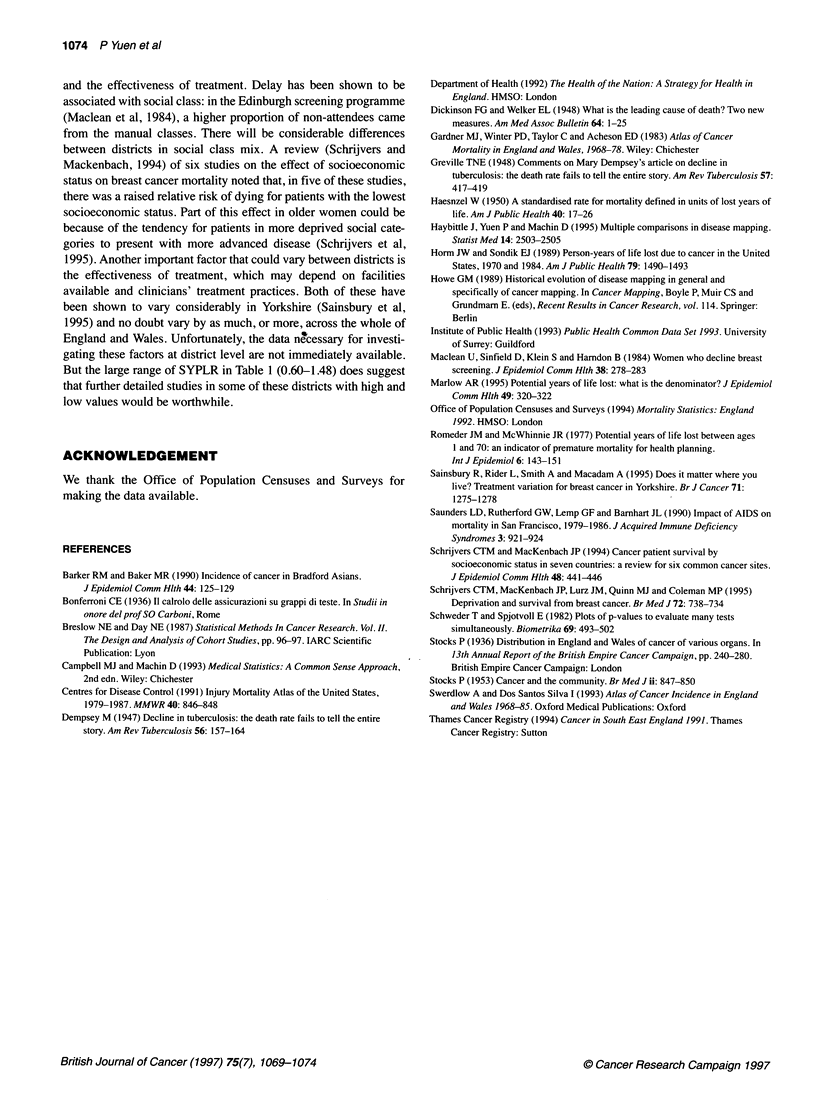# Geographical variation in the standardized years of potential life lost ratio (SYPLR) in women dying from malignancies of the breast in England and Wales.

**DOI:** 10.1038/bjc.1997.182

**Published:** 1997

**Authors:** P. Yuen, J. Haybittle, D. Machin

**Affiliations:** South West Thames Region NHS Executive, London, UK.

## Abstract

**Images:**


British Journal of Cancer (1997) 75(7), 1069–1074
© 1997 Cancer Research Campaign

### Geographical variation in the standardized years of potential life lost ratio (SYPLR) in women dying from malignancies of the breast in England and Wales

**P Yuen[1,2], J Haybittle[2] and D Machin[2]**

[1]South West Thames Region NHS Executive, 40 Eastbourne Terrace, London W2 3QR, UK; [2]MRC Cancer Trials Office, 5 Shaftesbury Road, Cambridge CB2 2BW, UK

**Summary** Geographical variation in the standardized years of potential life lost ratio (SYPLR) in women aged 20–74 dying from breast cancer has been mapped by local authority district in England and Wales and compared with the variation as described by the standardized mortality ratio (SMR). The geographical distribution of areas of low and high SMRs is similar to that observed some 15 years earlier, showing an increase from north to south. In contrast, the pattern of SYPLRs shows a less obvious trend. Years of life lost because of breast cancer mortality were particularly high in eight of the 401 county districts (SYPLR 1.22–1.48) and particularly low in nine (0.60–0.86). Mapping SYPLRs for breast cancer gives a different pattern from mapping SMRs, and one which is possibly more appropriate for targeting resources in circumstances in which death at younger age will have major social consequences. Care is required in interpreting maps indicating high and low rates in the presence of multiple comparisons. However, the range of values observed is suggestive of the need to investigate districts with contrasting values of SYPLR with respect to the inter-relationships between sociodemographic characteristics, duration of symptoms, clinical presentation and treatment efficacy.

**Keywords:** breast cancer; geographical variation; mortality; years lost

In England, the prevention and early detection of breast cancer in women is one of the five key areas for the Health of the Nation, an initiative put forward by the British government with the strategic goal of improving health through prevention and promotion (Department of Health, 1992). In England and Wales, 1 in 12 women develops breast cancer and, in 1988 alone, almost 27 000 new cases were diagnosed. This is approximately double the number of new cases of the next most common tumours, i.e. colorectal and lung, which affect both sexes (Thames Cancer Registry, 1994). Of the 13 663 women who died from breast cancer in 1992, 60% were under 75 years of age (Office of Population Censuses and Surveys, 1994).

In setting up objectives and target outcome for cancer as one of the key areas for Health of the Nation action, mortality and cancer incidence data were used to obtain age-standardized indicators However, there has been some concern over the use of the standardized mortality ratio (SMR) in this context.

Thus, the use of years of potential life lost (YPL) has been proposed (Dempsey, 1947; Greville, 1948; Haesnzel, 1950). This measure provides an indicator which gives more weight to deaths occurring at younger ages, thus emphasizing the impact of premature mortality (Romeder and McWhinnie, 1977). Such a measure reflects, in some sense, consequent social, family and economic burdens. It has been employed to investigate variation in accidents (Centres for Disease Control, 1991) and AIDS/HIV (Saunders et al, 1990) as well as cancer (Stocks, 1953; Horm and Sondik, 1989).

*Received 22 July 1996*
*Revised 22 July 1996*
*Accepted 30 September 1996*

*Correspondence to*: D Machin

Mapping of disease indicators has always been an important tool for epidemiologists, public health physicians and health care planners, especially when examining the geographic variation in cancers (Howe, 1989; Stocks, 1936) for clues to aetiology or to facilitate allocation of resources.

In this paper, we use the standardized years of potential life lost ratio (Haesnzel, 1950) (SYPLR), denoted the SYLL ratio by Marlow (1995), as a summary measure of the impact of premature mortality from breast cancer in women aged 20–74 years and investigate its geographical variation in England and Wales among 403 local authority districts. This age group was chosen as death from breast cancer in women under 20 is rare and the majority of women remain independent (healthwise) up to the age of 75. We also examine geographic variation among these districts using the SMR and compare this map with that of the 1960s given by Gardner et al (1983).

### MATERIALS AND METHODS

### The data

The mortality data, which relate to the resident population of England and Wales, were provided by the Office of National Statistics (ONS) and refer to underlying cause of death on death certificates for the years 1988–92. This dataset gives the number of deaths per year by tumour site and age in the 403 local authority districts. Districts were chosen that were small enough to detect subtle trends but, because of the paucity of deaths, the analysis was carried out by merging the 5 years of data. The alternative of grouping into larger geographical areas, on an annual basis, would provide numerical stability but a loss of sensitivity to detect geographical patterns. Population data for the districts consisted of estimates for each of the years 1988–92.

### Standardized mortality ratio (SMR) and standardized years of potential life lost ratio (SYPLR)

The SMR for female breast cancer deaths among women aged 20–74 in each local authority district was calculated using the conventional method (Campbell and Machin, 1993). The number of expected deaths ($E_j$) in the $j$th 5-year age group is given by $E_j = n_j D_j / N_j$, where $D_j$ is the total number of deaths in age group $j$ in the standard population (England and Wales), $N_j$ is the corresponding female population and $n_j$ is the district's population in the $j$th age group. If $d_j$ is the number of deaths in age group $j$ in the district population then:

$$\text{SMR} = \frac{\Sigma d_j}{\Sigma E_j}$$

where the summation is from 1 to $J$ and, in this study, 1 is the age group 20–24 years and $J$ the age group 70–74 years.

The years of potential life lost (YPL) is defined as the number of years of life lost as a consequence of each death occurring before a predetermined age. Thus, if a death occurs at age $k$ to $k + 4$ in the $j$th 5-year age group and this is before a particular age $c$, then the years of life lost is $a_j = c-(k + 2.5)$. The 2.5 is added to $k$ to indicate that the age of death of an individual within a 5-year age group is taken as being in the middle of the age group. In our study, $c = 75$ years.

The observed years of potential life lost is then given by YPL = $\Sigma a_j d_j$, while the expected values are $\Sigma a_j E_j$ and

$$\text{SYPLR} = \frac{\Sigma a_j d_j}{\Sigma a_j E_j}$$

Assuming that the standard population is very large, so that sampling errors in $\Sigma E_j$ can be ignored, and that $d_j$ follows a Poisson distribution, we have for the variances:

$$\text{Var(SMR)} = \text{Var}\left(\frac{\Sigma d_j}{\Sigma E_j}\right) = \frac{\Sigma \text{ Var } (d_j)}{(\Sigma E_j)^2} = \frac{\Sigma d_j}{(\Sigma E_j)^2}$$

and

$$\text{Var(SYPLR)} = \text{Var}\left(\frac{\Sigma a_j d_j}{\Sigma a_j E_j}\right) = \Sigma a_j^2 \frac{\text{Var } (d_j)}{(\Sigma a_j E_j)^2} = \frac{\Sigma a_j^2 d_j}{(\Sigma a_j E_j)^2}$$

For testing the differences of the SMRs and SYPLRs from unity or for the construction of confidence intervals, a logarithmic transformation is preferable to correct for skewness in the statistical distributions. It can be shown that:

$$\text{Var (log SMR)} = \frac{\text{Var (SMR)}}{(\text{SMR})^2}$$

and the standard error s.e. equals its square root. The test statistic for the SMR is

$$z_{SMR} = \frac{\text{log (SMR)}}{\text{s.e. [log(SMR)]}}$$

which can be compared with a normal deviate. Similarly, the test statistic for the SYPLR is:

$$z_{SYPLR} = \frac{\text{log (SYPLR)}}{\text{s.e. [log(SYPLR)]}}$$

### Colour coding of the maps

To permit comparisons over time, colour coding of maps and the convention for statistical significance were chosen to be the same as in Gardner et al (1983). Thus, six types of area are shaded on the maps according to the following scheme:
For districts with ratios greater than unity and the value of the test statistic is:

(1) ≥1.96 and ranked in the top tenth – dark red;
(2) ≥1.96 but not in the top tenth – mid-red;
(3) <1.96 but ranked in the top tenth – light red.

For districts with ratios less than unity and the absolute value of the test statistic is:

(1) <1.96 but ranked in the bottom tenth – light green;
(2) ≥1.96 but not in the bottom tenth – mid-green;
(3) ≥1.96 and in the bottom tenth – dark green.

All other areas and also two other areas, City of London (three deaths) and Isles of Scilly (one death), are left unshaded and appear white. City of London and Isles of Scilly are also omitted from the statistical testing. All other districts report 15 or more deaths.

### Multiple comparisons

The colour coding of the map uses a cut off of $z = 1.96$ or, equivalently, a test size or α-value of 0.05. It is recognized that, for comparisons within any map in this paper, 401 independent comparisons are made. As a consequence, using the procedure for colouring as described above there will be some spuriously significant districts of both low and high rates. One possibility is to compensate for this by use of the Bonferroni correction (Bonferroni, 1936; Campbell and Machin, 1993), which essentially replaces α by $\alpha_{\text{Bonferroni}}$, which is calculated from $(1 - \alpha_{\text{Bonferroni}})^n = 1 - \alpha$ and $n$ is the number of comparisons made. This equation leads to $\alpha_{\text{Bonferroni}} \approx \alpha/n$ and, for $\alpha = 0.05$ and $n = 401$, this gives $\alpha_{\text{Bonferroni}} = 0.05/401{=}0.00012$. Thus, for statistical significance at the nominal 5% level, a $P$-value <0.00012 or $z > 3.84$ is required.

An alternative approach first calculates the $P$-values for the $n$ comparisons involved (Schweder and Spjotvoll, 1982; Haybittle et al, 1995). These values are ranked and then plotted against the cumulative number of tests conducted. The slope of the straight line drawn through the origin and passing through the points in the left hand and central portion of this plot indicates the number of *true* null hypotheses ($m$), which will be less than or equal to $n$. This leads to the use of $\alpha_{ss} = 0.05/m$ in place of $\alpha_{\text{Bonferroni}} = 0.05/n$.

### RESULTS

Gardner et al (1983) concluded from their all-ages map for the SMR for breast cancer for 1968–78 that the main areas of low mortality were in the north of the country, including Lancashire, Yorkshire and Durham. In contrast, the south east and Midlands,

© Cancer Research Campaign 1997

**Figure 1** Standardized mortality ratios (SMR) from breast cancer, women aged 20–74 years, in local authority districts of England and Wales 1988–92

**Figure 2** Standardized years of potential life-lost ratios (SYPLR) from breast cancer, women aged 20–74 years, in local authority districts of England and Wales, 1988–92

**Figure 3** Age distribution of breast cancer death rates (1988–92) in two districts, Wear Valley and Rochester upon Medway

**Figure 4** Plot of $C_p$ vs 1-$P$ for the SYPLR from breast cancer, women aged 20–74 years, in 401 local authority districts of England and Wales, 1988–92

with occasional exceptions, had areas with high rates. Repeating these calculations of the SMR for the same (all) age groups but now on the 403 districts, and for the data of some 15 years later, resulted in a very similar pattern. A high proportion of the districts coloured some shade of green are in the north of the country, while all those with the darkest shade of red appear to the south-east of a line drawn from Whitby, North Yorkshire, to Plymouth, Devon.

However, to enable a more direct comparison of the distribution of the SMR compared with the SYPLR, the SMRs for the 20–74 years age group were calculated. The resulting map is shown in Figure 1. A test of heterogeneity (Breslow and Day, 1987) was significant ($\chi^2$ = 507.9, d.f. = 400; $P$ < 0.001), indicating the presence of some real differences between districts. All the districts coloured dark red still lie to the south-east of the Whitby–Plymouth line, but the low values in the north are not as numerous as in the all-ages map: only five coloured dark green compared with 12 on the all-ages map.

The variation in the SYPLR is mapped in Figure 2 and, again, a test of heterogeneity was significant ($P$ < 0.002). The districts shaded dark red are now less in the south-east, whereas there are six (Wear Valley, Trafford, Crewe and Nantwich, Newcastle under Lyme, South Herefordshire and Swansea) in the area to the north-west of the Whitby–Plymouth line. Of the six districts coloured dark red in Essex, Kent and Sussex in Figure 1, only two, Brentwood and Sevenoaks, remain dark red in Figure 2. No overall pattern now emerges.

As an example of how differences between the SMR and the SYPLR arise, Figure 3 shows the age distribution of the female breast cancer death rates in two districts, Rochester upon Medway and Wear Valley. The former has a high SMR (1.31) and a value of $z_{SMR}$ = 3.18 but a lower SYPLR (1.18) which is not significant ($z_{SYPLR}$ = 1.55). The rate is higher in Rochester upon Medway than in Wear Valley in the 60–74 years age group in which the deaths contributed relatively less years of life lost, and lower in the 45–59 and 20–44 years age groups, in which the contribution to YPL is greater. As a result the SYPLR in Wear Valley is high (1.42) with $z_{SYPLR}$ = 2.37, while the SMR is only 1.13 with $z_{SMR}$ = 1.00.

In some of the districts where $z \geq 1.96$ and $P \leq 0.05$, the high or low ratios could have arisen by chance in the 401 comparisons. A Bonferroni correction would replace the conventional $\alpha$ = 0.05 by $\alpha_{Bonferroni}$ = 0.00012, as we have indicated in the Methods section. There are no districts in which the SYPLR reaches this significance level. However, in certain circumstances, the Bonferroni procedure may be too conservative. We have therefore followed Schweder and Spjotvoll (1982) and plotted the cumulative number of $P$-values, $C_p$, against (1-$P$). The slope of the straight line drawn in Figure 4 is approximately 384, which, if taken as the number of 'true' null hypotheses, suggests that there could be 17 districts in which differences in SYPLR might not have arisen entirely by chance.

The 17 districts with the largest values of $z$ are listed in Table 1 and are mapped in Figure 5. They form no particular pattern. Only two districts, Bradford and Leeds, have a common boundary. Schweder and Spjotvoll (1982) say that their $P$-value plot '... is primarily intended for informal inference and it is difficult to make exact probability statements.' As an approximate test of significance they recommend, in cases such as this, using $\alpha_{ss}$ = 0.05/384 = 0.00013, which is only slightly higher than the conventional Bonferroni figure and would still result in no districts with statistically significant SYPLR. In the corresponding SMR calculations, the $z$-value for Bradford would just achieve significance on this basis, the breast cancer mortality being lower than expected (SMR = 0.79).

### DISCUSSION

Mortality data have long been used in addressing public health problems. Analysis of such data usually results in summary statistics such as rates, proportions, standardized mortality ratios SMRs and age-standardized rates. These indicators have been used in setting up public health priorities and in monitoring the health of the population. However, they often fail to address temporal changes in mortality and, in particular, they are dominated by elderly deaths. Thus more refined mortality measures (are needed) to give a more accurate picture, by weighting the deaths at younger ages to emphasise the impact of premature mortality.

© Cancer Research Campaign 1997

**Table 1** Seventeen local authority districts in the UK: the highest values of $z_{SYPLR}$ categorized as SYPLR greater or less than unity

| District | SYPLR | $z_{SYPLR}$ | SMR | $z_{SMR}$ | No.of deaths |
|---|---|---|---|---|---|
| *SYPLR > 1* | | | | | |
| Bassetlaw (Nottinghamshire) | 1.43 | 3.16 | 1.23 | 2.13 | 109 |
| Great Grimsby (Humberside) | 1.44 | 3.02 | 1.32 | 2.71 | 97 |
| Brentwood (Essex) | 1.42 | 2.78 | 1.36 | 2.91 | 89 |
| Corby (Northamptonshire) | 1.48 | 2.62 | 1.50 | 3.21 | 63 |
| Teignbridge (Devon) | 1.29 | 2.44 | 1.19 | 1.97 | 128 |
| Trafford (Greater Manchester) | 1.22 | 2.42 | 1.15 | 1.99 | 210 |
| Swansea (West Glamorgan) | 1.23 | 2.39 | 1.13 | 1.70 | 190 |
| Wear Valley (Durham) | 1.42 | 2.37 | 1.13 | 1.00 | 65 |
| *SYPLR < 1* | | | | | |
| Bradford (West Yorkshire) | 0.78 | 3.43 | 0.79 | 3.92 | 288 |
| Monmouth (Gwent) | 0.60 | 2.98 | 0.81 | 1.54 | 65 |
| Huntingdonshire (Cambridgeshire) | 0.68 | 2.86 | 0.75 | 2.33 | 77 |
| Leeds (West Yorkshire) | 0.86 | 2.85 | 0.89 | 2.73 | 517 |
| Ogwr (Mid Glamorgan) | 0.69 | 2.84 | 0.77 | 2.74 | 88 |
| Thamesdown (Wiltshire) | 0.73 | 2.64 | 0.86 | 1.55 | 111 |
| Kensington and Chelsea (London) | 0.71 | 2.48 | 0.78 | 2.25 | 82 |
| Kerrier (Cornwall and Isles of Scilly) | 0.69 | 2.39 | 0.74 | 2.32 | 60 |
| Luton (Bedfordshire) | 0.74 | 2.30 | 0.77 | 2.50 | 88 |

**Figure 5** Map highlighting the 17 districts in England and Wales with the highest absolute values of $z_{SYPLR}$. Red, SYPLR > 1; green, SYPLR < 1.

The use of years of life lost arose in the late 1940s to answer questions such as 'What is the leading cause of death?' (Dickinson and Welker, 1948). Since then, the methodology has evolved and has been widely used in the United States. In England and Wales, the annual all-cause YLL has been published by the OPCS regularly. Selected cause-specific YLL and age-standardized rates per 100 000 residents by health districts have been produced and published by the Department of Health in the Public Health Common Dataset since 1990 (Institute of Public Health, 1993).

SYPLR as an index for premature mortality complements mortality indicators such as SMR and age-standardized rates. It is simple to understand and can be useful in defining health priorities and programmes for the prevention of premature deaths and targeting health education to those in need. Mapping the SYPLR has the added advantage of revealing the geographic pattern of populations at risk.

An important question concerns how much of the variation between districts shown in the maps is random. Use of the Bonferroni correction, even when modified according to Schweder and Spjotvoll (1982), would suggest that practically all could be attributed to sampling variation. This is probably a very conservative conclusion as the plot in Figure 4 indicates that there are approximately 17 null hypotheses which should be rejected. Hence, our decision to draw attention to the districts in Table 1 that had the highest values of $z_{SYPLR}$. However, as reported elsewhere (Haybittle et al, 1995), some of the differences in these selected districts will have arisen purely by chance, while we are missing others where there exist 'true' differences which could be of practical importance in health terms.

If we consider the districts in Table 1, then some of the excesses and deficits of years lost could, at least in part, be explained by variations in incidence. Unfortunately, for our purpose, the most recent atlas of cancer incidence in England and Wales (Swerdlow and dos Santos Silva, 1993) analyses by county rather than by district. There is, however, some relevant information for Bradford, which topped the rankings in both $z_{SMR}$ and $z_{SYPLR}$. Some 10% of the population is of Asian origin and the standardized registration ratio for female breast cancer in the Asian population is 44 (Barker and Baker, 1990). This implies an overall reduction of 4–5% (10% of 44) in incidence, which could account for part of the reduction in YPL in this district.

Other factors that could contribute to the variation in SYPLR are differences in delay before seeking treatment, clinical presentation and the effectiveness of treatment. Delay has been shown to be associated with social class: in the Edinburgh screening programme (Maclean et al, 1984), a higher proportion of non-attendees came from the manual classes. There will be considerable differences between districts in social class mix. A review (Schrijvers and Mackenbach, 1994) of six studies on the effect of socioeconomic status on breast cancer mortality noted that, in five of these studies, there was a raised relative risk of dying for patients with the lowest socioeconomic status. Part of this effect in older women could be because of the tendency for patients in more deprived social categories to present with more advanced disease (Schrijvers et al, 1995). Another important factor that could vary between districts is the effectiveness of treatment, which may depend on facilities available and clinicians' treatment practices. Both of these have been shown to vary considerably in Yorkshire (Sainsbury et al, 1995) and no doubt vary by as much, or more, across the whole of England and Wales. Unfortunately, the data necessary for investigating these factors at district level are not immediately available. But the large range of SYPLR in Table 1 (0.60–1.48) does suggest that further detailed studies in some of these districts with high and low values would be worthwhile.

### ACKNOWLEDGEMENT

We thank the Office of Population Censuses and Surveys for making the data available.

### REFERENCES

Barker RM and Baker MR (1990) Incidence of cancer in Bradford Asians. *J Epidemiol Comm Hlth* **44**: 125–129

Bonferroni CE (1936) Il calrolo delle assicurazioni su grappi di teste. In *Studii in onore del prof SO Carboni*, Rome

Breslow NE and Day NE (1987) *Statistical Methods In Cancer Research. Vol. II. The Design and Analysis of Cohort Studies*, pp. 96–97. IARC Scientific Publication: Lyon

Campbell MJ and Machin D (1993) *Medical Statistics: A Common Sense Approach*, 2nd edn. Wiley: Chichester

Centres for Disease Control (1991) Injury Mortality Atlas of the United States, 1979–1987. *MMWR* **40**: 846–848

Dempsey M (1947) Decline in tuberculosis: the death rate fails to tell the entire story. *Am Rev Tuberculosis* **56**: 157–164

Department of Health (1992) *The Health of the Nation: A Strategy for Health in England*. HMSO: London

Dickinson FG and Welker EL (1948) What is the leading cause of death? Two new measures. *Am Med Assoc Bulletin* **64**: 1–25

Gardner MJ, Winter PD, Taylor C and Acheson ED (1983) *Atlas of Cancer Mortality in England and Wales, 1968–78*. Wiley: Chichester

Greville TNE (1948) Comments on Mary Dempsey's article on decline in tuberculosis: the death rate fails to tell the entire story. *Am Rev Tuberculosis* **57**: 417–419

Haesnzel W (1950) A standardised rate for mortality defined in units of lost years of life. *Am J Public Health* **40**: 17–26

Haybittle J, Yuen P and Machin D (1995) Multiple comparisons in disease mapping. *Statist Med* **14**: 2503–2505

Horm JW and Sondik EJ (1989) Person-years of life lost due to cancer in the United States, 1970 and 1984. *Am J Public Health* **79**: 1490–1493

Howe GM (1989) Historical evolution of disease mapping in general and specifically of cancer mapping. In *Cancer Mapping*, Boyle P, Muir CS and Grundmarn E. (eds), *Recent Results in Cancer Research, vol.* 114. Springer: Berlin

Institute of Public Health (1993) *Public Health Common Data Set 1993*. University of Surrey: Guildford

Maclean U, Sinfield D, Klein S and Harndon B (1984) Women who decline breast screening. *J Epidemiol Comm Hlth* **38**: 278–283

Marlow AR (1995) Potential years of life lost: what is the denominator? *J Epidemiol Comm Hlth* **49**: 320–322

Office of Population Censuses and Surveys (1994) *Mortality Statistics: England 1992*. HMSO: London

Romeder JM and McWhinnie JR (1977) Potential years of life lost between ages 1 and 70: an indicator of premature mortality for health planning. *Int J Epidemiol* **6**: 143–151

Sainsbury R, Rider L, Smith A and Macadam A (1995) Does it matter where you live? Treatment variation for breast cancer in Yorkshire. *Br J Cancer* **71**: 1275–1278

Saunders LD, Rutherford GW, Lemp GF and Barnhart JL (1990) Impact of AIDS on mortality in San Francisco, 1979–1986. *J Acquired Immune Deficiency Syndromes* **3**: 921–924

Schrijvers CTM and MacKenbach JP (1994) Cancer patient survival by socioeconomic status in seven countries: a review for six common cancer sites. *J Epidemiol Comm Hlth* **48**: 441–446

Schrijvers CTM, MacKenbach JP, Lurz JM, Quinn MJ and Coleman MP (1995) Deprivation and survival from breast cancer. *Br Med J* **72**: 738–734

Schweder T and Spjotvoll E (1982) Plots of p-values to evaluate many tests simultaneously. *Biometrika* **69**: 493–502

Stocks P (1936) Distribution in England and Wales of cancer of various organs. In *13th Annual Report of the British Empire Cancer Campaign*, pp. 240–280. British Empire Cancer Campaign: London

Stocks P (1953) Cancer and the community. *Br Med J* **ii**: 847–850

Swerdlow A and Dos Santos Silva I (1993) *Atlas of Cancer Incidence in England and Wales 1968–85*. Oxford Medical Publications: Oxford

Thames Cancer Registry (1994) *Cancer in South East England 1991*. Thames Cancer Registry: Sutton

*© Cancer Research Campaign 1997*